# Social inequality and age-specific gender differences in overweight and perception of overweight among Swedish children and adolescents: a cross-sectional study

**DOI:** 10.1186/s12889-015-1985-x

**Published:** 2015-07-09

**Authors:** JS van Vliet, PA Gustafsson, K Duchen, N Nelson

**Affiliations:** Department of Clinical and Experimental Medicine, Division of Pediatrics, Faculty of Health Sciences, Linköping University, Linköping, SE-58185 Sweden; Department of Clinical and Experimental Medicine, Division of Child and Adolescent Psychiatry, Faculty of Health Sciences, Linköping University, Sweden and County Council of Östergötland, Linköping, SE-58185 Sweden; Department of Pediatrics, University Hospital, County Council of Östergötland, Linköping, SE-58185 Sweden; Quality and Patient Safety, Karolinska University Hospital, Stockholm, SE-17176 Sweden

**Keywords:** Social inequality, Overweight, Obesity, Perception of overweight, Childhood, Adolescence

## Abstract

**Background:**

Overweight among children and adolescents related to social inequality, as well as age and gender differences, may contribute to poor self-image, thereby raising important public health concerns. This study explores social inequality in relation to overweight and perception of overweight among 263 boys and girls, age 7 to 17, in Växjö, Sweden.

**Methods:**

Data were obtained through a questionnaire and from physical measurements of height, weight and waist circumference [WC]. To assess social, age and gender differences in relation to overweight, the independent sample t- and chi-square tests were used, while logistic regression modeling was used to study determinants for perception of overweight.

**Results:**

Social inequality and gender differences as they relate to high ISO-BMI [Body Mass Index for children] and WC were associated with low maternal socioeconomic status [SES] among boys < 13 years [mean age = 10.4; n = 65] and with low paternal education level among boys ≥ 13 years [mean age = 15.0; n = 39] [p < 0.05]. One suggested explanation for this finding is maternal impact on boys during childhood and the influence of the father as a role model for adolescent boys. The only association found among girls was between high ISO-BMI in girls ≥ 13 years [mean age = 15.0; n = 74] and low paternal occupational status. Concerning perception of overweight, age and gender differences were found, but social inequality was not the case. Among boys and girls < 13 years, perception of overweight increased only when overweight was actually present according to BMI or WC [p < 0.01]. Girls ≥ 13 years [mean age = 15.0] were more likely to unrealistically perceive themselves as overweight or “too fat,” despite factual measurements to the contrary, than boys [p < 0.05] and girls < 13 years [mean age = 10.4; n = 83] [p < 0.001].

**Conclusions:**

The association between social inequality and overweight in adolescence in this study is age- and gender-specific. Gender differences, especially in perception of overweight, tend to increase with age, indicating that adolescence is a crucial period. When planning interventions to prevent overweight and obesity among children and adolescents, parental SES as well as age and gender-specific differences in social norms and perception of body weight status should be taken into account.

## Background

The increasing prevalence of overweight among children and adolescents related to social inequality has become a major concern in the field of public health in many countries, including Sweden [1–3]. Since 1990, the dominant pattern seen in developed countries is one in which greater socioeconomic disadvantage is associated with higher prevalence of overweight and obesity among children, matching the pattern for adults [4, 5]. Moreover, the 2005/6 WHO Health Behavior in School-aged Children Survey [HBSC] reports a significant negative association between family affluence and self-reported overweight and obesity, while children from more deprived families are more likely to be obese or overweight, especially in Western Europe [6, 7]. Most studies focus on familial socioeconomic status [SES], a classification based on income, parental education, or occupational status [1–7]. In Swedish health surveys, income is rarely included in SES estimation since the characteristics of the welfare state differ radically from other countries and the welfare transfer ration system make this variable less reliable [8]. When a distinction is made between paternal and maternal SES in relation to prevalence of overweight, lower maternal SES is found to increase the risk for overweight and especially for obesity among younger children. The suggested explanation for the relationship between obesity among younger children and lower maternal SES may relate to lifestyle characteristics of mother and child [9].

The prevalence of childhood overweight and obesity is still rapidly increasing worldwide, including in Sweden [3, 10]. Since 1980, the prevalence of overweight and obesity has increased remarkably in developed countries. In Sweden, 24.7 % [21.1–28.7] of boys and 23.3 % [19.7–27.5] of girls <20 years were overweight or obese in 2013, compared with a prevalence of 16.9 % [16.1–17.7] in boys and 16.2 % [15.5–17.1] in girls in 1980 in developed countries [11]. At the same time, there is evidence that suggest decreasing prevalence of overweight in girls [12]. In the Oslo Health Study [HUBRO], SES is associated with overweight and obesity only in boys and only in relation to parental level of education [13]. Lien et al. suggest that social norms concerning weight and perception of body weight status among girls cut across SES, whereas among boys, awareness of social norms regarding weight is mainly associated with parental education, perhaps related to parental knowledge and family role models [13].

Children of both sexes from lower SES households were found to underestimate overweight compared with their respective counterparts, and may therefore lack motivation to avoid further weight gain [14]. The already higher prevalence of overweight in these groups is therefore unlikely to decrease. At the same time, girls in particular overestimate their weight, which is a risk factor for unhealthy weight-control behaviors [14]. Thus, from the public health perspective, regardless of other known risk factors for development of obesity [15], it is important to understand body and weight perception among adolescents in relation to actual body and weight measurements, both to avoid further weight gain in case of underestimation and to prevent unhealthy weight-control behaviors in case of overestimation.

We hypothesize that the pubertal developmental process may influence the relationship between SES and overweight/obesity, as well as perception of body weight status among children and adolescents. Puberty is an important period of transition between childhood and adulthood. In Scandinavian countries, mean age for onset of puberty among girls is 10.9 [8.7 - 13.1, +/−2SD] and among boys 11.8 [9.9 - 13.8, +/−2SD] [16]. Important changes during puberty include increased growth in height and sexual maturation, as well as changes in body composition among both boys and girls. Hallmarks of pubertal development are peak height velocity occurring at the mean age of 13.5 in boys and 11.5 in girls [17], as well as menarche in girls at the mean age of 13.4 [11.2 – 15.7, +/−2SD] [16]. During this transition period and the pubertal maturation process, a possible crucial time for changing behaviors and attitudes occurs at age 13 when, as teenagers, Swedish children leave primary school to begin attending secondary school.

The aim of this paper is to report how gender, age and parental SES are associated to the risk for developing overweight and obesity in boys and girls in the two age groups 7–13 and 13–17 years. Furthermore, we describe whether social inequality, as it relates to perception of body weight status among these boys and girls, may be associated to age and gender differences in the prevalence of overweight/obesity. Increasing knowledge and understanding about how social inequality relates to overweight and perception of overweight in young people may improve health strategies and contribute to prevention of future weight-related disorders and diseases.

## Methods

### Participants

In a cross-sectional study, children and adolescents aged 7 to 17 from the second to seventh grade in primary school and from seventh to ninth grade in secondary school in Växjö, Sweden, were invited to participate in the measurements consisting of a written questionnaire and anthropometry [N = 391]. Their parents were invited by letter to supply with additional information in a written questionnaire of which the background variables on SES were most relevant for the actual study. Parents and children were informed by letter and at special organized occasions at the schools, for parents in the evening hours, for children during school hours. Written consent was obtained from all parents as well as from all children from the seventh grade, i.e. about twelve years and upwards. Sixty seven percent [n = 263] of the children participated in this study during the autumn of 2010. Participation rates among boys and girls were 60 % [N = 175] and 73 % [N = 216], respectively. Among their parents, 85 % [N = 395] returned the questionnaire.

### Materials and methods

Self-reports on background variables as parental SES pertaining to level of paternal and maternal education and occupational status, were obtained as a part of a written questionnaire that was filled out by the parents at home and returned by mail to the research group at Linköping University. Participating boys and girls were asked to complete a written questionnaire during school hours in the classroom environment. For this part of the study, a series of 5 questions were posed from the international WHO survey on Health Behavior in School-aged Children [HBSC] to be able to compare with results from other countries [18]. To assess perception of the own body weight status we used a HBSC-question that was more specific, “Do you think your body is…”. Response categories were based on the five-point Likert scale and ranged from “far too thin” to “far too fat”.

In addition to the self-reported questionnaire data, the school nurse obtained physical measurements of height, weight and waist circumference from each individual in private using calibrated school equipment. Weight was measured to the nearest 100 g, using an electronic scale. Height was measured to the nearest 0.5 cm using a wall-mounted wooden ruler [Hultafors, Hultafors Group Sverige AB, Bollebygd, Sweden]. The children were weighed wearing light clothing, t-shirt and trousers, but without shoes and belts. Waist and hip circumference were obtained using a yielding measuring tape. Hip circumference [cm] was measured at the widest point to the nearest 0.1 cm. Waist circumference [cm] was measured to the nearest 0.1 cm midway between the tenth rib and the iliac crest. All measurements were obtained in school during school hours.

### Comparisons and data analysis

In addition to directly obtained physical measurements, we calculated body mass index [BMI = weight/height^2^]. BMI of the adolescents was categorized using the age- and gender-specific ISO-BMI cut-off points for overweight [ISO-BMI 25] and obesity [ISO-BMI 30] as defined by Cole et al. [19]. As in their study, we focused on overweight and perception of overweight, using three categories: obesity, overweight and non-overweight. To determine the age- and gender-specific cut-off points for overweight according to WC, we used the percentiles developed by McCarthy et al. [20].

From the answers to the question pertaining to perception of body weight status, we defined perception of overweight as a score of four or higher on the Likert scale [i.e. “too fat” or “far too fat”]. Perception of body weight status was dichotomized into non-overweight vs. perception of overweight, since this study focuses on perception of overweight. In the analysis, we combined measured overweight by ISO-BMI or WC and perception of overweight into one category and defined misconception 1 as perception of overweight without measured overweight and misconception 2 as perception of non-overweight despite measured overweight.

We classified children into two age groups with a cut-off point at 13 years, with younger children defined as age 7 to 12.99 years and older children as ≥ 13 years. The age 13 cut-off thereby enabled us to compare children in childhood [mean age 10.4 ± 1.5 years; n = 148] with those in adolescence [mean age 15.0 ± 0.9 years; n = 113], while also allowing a comparison between children attending primary school and teenagers attending secondary school. Mean age at menarche among girls in this study was 12.6 ± 1.2 years [n = 78].

Parental socioeconomic status [SES] was classified according to either level of education or occupational status, as defined by Statistics Sweden [21]. Level of education for mothers and fathers, respectively, was divided into two categories: those who pursued studies beyond secondary school at college or university were defined as having a higher level of education, while those who left school after graduating from a vocationally oriented secondary school were defined as having a lower level. Regarding occupational status, blue-collar workers were classified as having lower occupational status and white collar workers as having higher occupational status, according to the international classification system used by Statistics Sweden [21]. It is important to remember that level of education and occupational status are not completely independent of one another. A Spearman correlation coefficient between level of education and occupational status among mothers was 0.61 [p = 0.042; n = 299] and among fathers 0.56 [p = 0.045; n = 284]. Within families, between mothers and fathers, the correlation coefficient for level of parental education was 0.42 [p = 0.053; n = 292] and for parental occupational status 0.35 [p = 0.056; n = 306].

The collected information was coded and entered into the SPSS 22.0 statistics program. To compare groups, the *t*-test was used for continuous variables and the chi-square test for non-parametric variables. We used univariate logistic regression modeling to study the determinants for overweight and perception of overweight, as well as logistic regression to assess odds ratios of perception of overweight. Logistic regression was also used to answer the question of the extent to which perception of overweight may vary with independent variables such as age, gender and socioeconomic status. Multiple regression modeling was used to adjust for measured overweight according to ISO-BMI and for age. Due to attrition, the number of subjects in separate analyses varied depending on the variables included. Level of significance was set at p < 0.05 for all analyses.

### Ethical considerations

The research herein was part of a larger research project, which was preceded by written information on the purpose of the research, information on voluntary participation, consent and confidentiality in five languages [Swedish, Arabic, Albanian, Somali and Serbo-Croatian] for all participating children and their families at the school. All families were also invited to information meetings with the research group. Written parental informed consent was obtained before the study from all parents and from all children from seventh grade [12–13 years old] upwards. The study was approved by the Regional Ethical Review Board in Linköping D-nr 07–182.

## Results

### Overweight and obesity

The prevalence of overweight/obesity among participants 7 to 17 years in this study was 14 % for boys [n = 106] and 15 % for girls [n = 157] when classified according to ISO-BMI. When classified according to WC the corresponding prevalence of overweight and obesity was 26 % for boys and 36 % for girls. No significant gender differences in prevalence of overweight/obesity were found according either to ISO-BMI classification or to WC.

Only among boys < 13 years [mean = 10.4 years, n = 65] did age- and gender-specific analyses show an association between low parental SES, particularly low maternal SES, and higher prevalence of overweight/obesity [Table 1]. In this group of boys, a high mean ISO-BMI and mean WC. were found to be associated with low maternal SES [p < 0.05]. Only lower paternal occupational status was related to high ISO-BMI in this group [p < 0.05]. When assessing the relationship between BMI /WC and parental SES, the findings were similar regarding the previous association between overweight/obesity and maternal SES in boys younger than 13 years [Table 2]. Among boys ≥ 13 years [mean = 15.1 years, n = 39], only an association between a high mean BMI and WC [p < 0.05] and low level of paternal education was found [Table 2].

**Table 1:** Prevalence of overweight/obesity according to ISO-BMI and WC for gender, age and parental SES

	Maternal Socioeconomic Status						Paternal Socioeconomic Status					
	Educational level			Occupational level			Educational level			Occupational level		
	Lower	Higher	P-value	Lower	Higher	P-value	Lower	Higher	P-value	Lower	Higher	P-value
**Boys < 13 years**	[n = 21]	[n = 36]		[n = 21]	[n = 42]		[n = 24]	[n = 30]		[n = 24]	[n = 36]	
Overweight/obese ISO-BMI*	33.3 %	5.6 %	0.006	38.1 %	4.8 %	0.001	25.0 %	6.7 %	0.060	29.2 %	5.6 %	0.012
Overweight/obese WC**	42.9 %	19.4 %	0.058	47.6 %	21.4 %	0.030	33.3 %	26.7 %	0.594	41.7 %	25.0 %	0.174
**Boys ≥ 13 years**	[n = 21]	[n = 15]		[n = 18]	[n = 20]		[n = 19]	[n = 16]		[n = 11]	[n = 24]	
Overweight/obese ISO-BMI*	9.5 %	13.3 %	0.720	11.1 %	10.0 %	0.911	15.8 %	6.3 %	0.377	9.1 %	12.5 %	0.769
Overweight/obese WC**	23.8 %	6.7 %	0.174	22.2 %	10.0 %	0.302	26.3 %	6.3 %	0.117	27.3 %	12.5 %	0.282
**Girls < 13 years**	[n = 37]	[n = 38]		[n = 27]	[n = 54]		[n = 39]	[n = 34]		[n = 28]	[n = 48]	
Overweight/obese ISO-BMI*	16.2 %	18.4 %	0.801	14.8 %	18.5 %	0.678	17.9 %	20.6 %	0.775	17.9 %	18.8 %	0.923
Overweight/obese WC**	45.9 %	34.2 %	0.300	48.1 %	33.3 %	0.196	43.6 %	41.2 %	0.835	46.4 %	37.5 %	0.445
**Girls ≥ 13 years**	[n = 29]	[n = 36]		[n = 20]	[n = 46]		[n = 36]	[n = 31]		[n = 23]	[n = 43]	
Overweight/obese ISO-BMI*	10.3 %	11.1 %	0.921	10.7 %	8.0 %	0.865	13.9 %	9.7 %	0.596	21.7 %	9.3 %	0.161
Overweight/obese WC**	37.9 %	25.0 %	0.262	30.0 %	28.3 %	0.886	27.8 %	35.5 %	0.498	39.1 %	30.2 %	0.465

*ISO-BMI = age and gender-specific body mass index [BMI] for children up to 18 years. Cut-off point for overweight/obesity according to ISO-BMI defined by Cole et al. [19]. **WC = Waist Circumference. Cut-off points for overweight/obesity according to WC percentiles produced by McCarthy et al. [20]

**Table 2:** Mean and standard deviations for BMI and WC according to gender, age and parental SES

	Maternal Socioeconomic Status						Paternal Socioeconomic Status					
	Educational level			Occupational level			Educational level			Occupational level		
	Lower	Higher	P-value	Lower	Higher	P-value	Lower	Higher	P-value	Lower	Higher	P-value
**Boys <13 years**	[n = 21]	[n = 36]		[n = 21]	[n = 42]		[n = 24]	[n = 30]		[n = 24]	[n = 36]	
BMI [kg/m^2^]*	19.3 ± 3.9	17.2 ± 2.9	0.019	20.1 ± 4.2	17.1 ± 2.3	0.000	18.8 ± 3.9	17.3 ± 3.1	0.117	18.7 ± 4.1	17.6 ± 2.9	0.199
WC [cm]**	66.9 ± 12.4	62.1 ± 7.4	0.073	68.5 ± 12.7	61.8 ± 6.6	0.008	65.3 ± 12.0	62.9 ± 8.1	0.401	65.6 ± 12.5	63.2 ± 7.3	0.349
**Boys ≥13 years**	[n = 21]	[n = 15]		[n = 18]	[n = 20]		[n = 19]	[n = 16]		[n = 11]	[n = 24]	
BMI [kg/m^2^]*	20.9 ± 2.3	19.5 ± 2.3	0.072	20.6 ± 2.5	19.9 ± 2.2	0.360	20.9 ± 2.4	19.3 ± 2.0	0.042	20.7 ± 2.3	19.9 ± 2.4	0.348
WC [cm]**	73.5 ± 5.5	71.2 ± 4.7	0.203	73.1 ± 5.5	72.1 ± 4.9	0.541	74.0 ± 5.9	70.5 ± 3.6	0.049	73.2 ± 6.0	71.9 ± 4.9	0.484
**Girls <13 years**	[n = 37]	[n = 38]		[n = 27]	[n = 54]		[n = 39]	[n = 34]		[n = 28]	[n = 48]	
BMI [kg/m^2^]*	18.0 ± 3.0	17.8 ± 4.1	0.745	18.4 ± 2.9	17.7 ± 3.7	0.397	18.2 ± 3.4	17.9 ± 3.8	0.718	18.3 ± 3.8	17.9 ± 3.4	0.618
WC [cm]**	62.9 ± 7.7	61.8 ± 10.2	0.600	63.8 ± 7.8	61.4 ± 9.2	0.248	63.1 ± 8.7	61.8 ± 9.4	0.543	62.6 ± 9.4	62.2 ± 8.8	0.885
**Girls ≥13 years**	[n = 29]	[n = 36]		[n = 20]	[n = 46]		[n = 36]	[n = 31]		[n = 23]	[n = 43]	
BMI [kg/m^2^]*	21.0 ± 3.3	20.6 ± 2.3	0.581	21.1 ± 3.2	20.4 ± 2.4	0.280	21.0 ± 3.0	20.6 ± 2.7	0.573	21.9 ± 3.1	20.4 ± 2.6	0.042
WC [cm]**	70.0 ± 6.6	69.6 ± 6.1	0.757	69.9 ± 5.6	69.0 ± 6.6	0.585	70.2 ± 6.6	69.1 ± 6.1	0.487	71.5 ± 6.5	68.9 ± 6.1	0.112

*BMI = body mass index [BMI]. **WC = Waist Circumference

We found no differences neither in the prevalence of overweight/obesity, nor in mean BMI or WC in relation to SES among girls < 13 years [mean age = 10.4; n = 83]. In girls ≥ 13 years [mean = 15.0 years, n = 74], however, high mean BMI and WC were found [p < 0.05] to be associated with low level of paternal occupational status, but not with paternal educational status [Table 2].

### Perception of overweight

A logistic regression model predicted that girls [n = 157] were at a 2.65 [CI 1.3-5.4; p < 0.01] times higher risk for perception of overweight than boys [n = 104]. We present separate regression models for boys and girls that provide odds ratios [OR] for determinants for perception of overweight [Table 3]. For boys, only measured overweight according to either ISO-BMI or WC was a significant determinant for perception of overweight [p < 0.01]. Adjusting for age ≥ 13 years showed that ISO-BMI and WC were still significant determinants. Separate regression models for measured overweight among boys younger and older than 13 years, respectively, revealed that the model could not be run for boys ≥ 13 years because of an empty cell in the model, implying the exact OR to be zero. Among younger boys, the OR for perception of overweight increased for both measured overweight according to ISO-BMI and to WC compared with all boys [p < 0.01] [Table 3]. Among boys, measured overweight according to WC was no longer a significant determinant for perception of overweight after adjusting for measured overweight according to ISO-BMI. Neither age nor parental SES was found to be related to perception of overweight in boys.

**Table 3:** Logistic univariate regression model predicting exact odds ratios [OR] for determinants of perception of overweight

					Adjusted for overweight ISO-BMI*		Adjusted for age ≥13 years	
			OR [CI] for perception of overweight	P-value	OR [CI] for perception of overweight	P-value	OR [CI] for perception of overweight	P-value
**Boys [n = 104]**	Overweight/obese ISO-BMI*		26.7 [6.2 - 114.7]	0.000			24.4 [5.6 – 106.7]	0.000
	Boys < 13 years [n = 65]		65.3 [9.4 - 454.2]	0.000				
	Boys ≥ 13 years [n = 39]		0.0^1]^					
	Overweight/obese WC**		8.8 [2.3 - 32.7]	0.001	1.8 [0.3 - 11.9]	0.547	7.6 [2.0 - 28.9]	0.003
	Boys < 13 years [n = 65]		14.9 [2.8 - 80.5]	0.002	2.0 [0.2 – 24.9]	0.587		
	Boys ≥ 13 years [n = 39]		0.0^1]^		0.0^1]^			
	**Age ≥ 13 years**		0.3 [0.1 - 1.6]	0.163	0.4 [0.7 - 2.4]	0.322		
	Maternal SES***	**higher education**	0.5 [0.1 – 1.7]	0.251	0.7 [0.1 – 3.2]	0.619	0.3 [0.1 – 1.3]	0.101
		**higher occupation**	0.5 [1.3 - 1.6]	0.212	1.1 [0.2 - 5.4]	0.926	0.4 [0.1 – 1.4]	0.153
	Paternal SES***	**higher education**	0.4 [0.1 – 1.4]	0.146	0.6 [0.1 – 3.6]	0.617	0.3 [0.1 – 1.2]	0.093
		**higher occupation**	0.4 [0.1 - 1.3]	0.122	0.8 [0.1 - 4.1]	0.743	0.4 [0.1 – 1.5]	0.152
**Girls [n = 157]**	Overweight/obese ISO-BMI*		6.8 [2.7 - 17.5]	0.000			20.4 [5.1 - 81.0]	0.000
	Girls < 13 years [n = 83]		66.3 [7.2 - 611.5]	0.000				
	Girls ≥ 13 years [n = 74]		5.7 [1.1 - 30.0]	0.039				
	Overweight/obese WC**		3.3 [1.5 - 7.2]	0.003	1.4 [0.5 – 4.0]	0.562	4.6 [1.9 – 11.2]	0.001
	Girls < 13 years [n = 83]		0.0^1]^		0.0^1]^			
	Girls ≥ 13 years [n = 74]		2.1 [0.7 - 5.9]	0.162	1.1 [0.3 – 3.9]	0.890		
	**Age ≥ 13 years**		5.5 [2.4 - 12.9]	0.000	13.9 [4.0 – 48.7]	0.000		
	Maternal SES***	**higher education**	1.3 [0.6 – 2.9]	0.515	1.2 [0.5 – 2.9]	0.621	1.2 [0.5 – 2.8]	0.724
		**higher occupation**	0.9 [0.4 - 2.1]	0.808	0.8 [0.3 – 2.0]	0.822	0.8 [0.3 – 2.1]	0.696
	Paternal SES***	**higher education**	1.2 [0.6 – 2.6]	0.645	1.2 [0.5 – 2.6]	0.707	1.2 [0.5 – 2.8]	0.663
		**higher occupation**	0.8 [0.3 - 1.7]	0.499	0.8 [0.3 – 1.8]	0.545	0.6 [0.3 – 1.5]	0.311

^1]^ Exact OR was zero because of one empty cell

*ISO-BMI = age and gender-specific body mass index [BMI] for children up to 18 years. Cut-off point for overweight/obesity according to ISO-BMI defined by Cole et al. [19]. **WC = Waist Circumference. Cut-off points for overweight/obesity according to WC percentiles produced by McCarthy et al. [20]. ***SES = Socioeconomic status regarding educational and occupational level respectively

For girls, measured overweight according to ISO-BMI and/or WC, as well as age ≥ 13 were significant determinants for perception of overweight [p < 0.01]. Similarly to the boys, the OR for perception of overweight when measuring overweight according to WC was no longer significant when adjusted for overweight according to ISO-BMI. However, the OR for perception of overweight increased among girls who were overweight according to ISO-BMI and WC when adjusted for age and showed that measured overweight was a stronger determinant in the younger age group of girls, <13 years [p < 0.01]. Age-specific models for girls confirmed this finding for perception of overweight in the presence of measured overweight according to ISO-BMI with ORs of 66.3 [CI 7.2-611.5; p < 0.001] and 5.7 [CI 1.1-30; p < 0.05] for the younger and older girls, respectively [p < 0.05]. Age ≥13 years as a single determinant for perception of overweight was found to be significant and the OR that age ≥ 13 years influences perception of overweight increased significantly when adjusted for measured overweight according to ISO-BMI [p < 0.001]. As in the case of boys, parental SES was not a significant determinant for perception of overweight in girls.

The results described above show that age and gender differences were associated with the prevalence of misconception 1. Girls ≥ age 13 significantly more often perceived themselves as overweight, despite measured non-overweight according to ISO-BMI and WC, than did boys of any age [p < 0.05] and girls < age 13 [p < 0.01] [Table 4]. No significant gender or age differences were found for misconception 2, where overweight was measured, but not perceived.

**Table 4:** Prevalence of misconception 1 and misconception 2 for boys and girls respectively

				**Boys**			**Girls**		
	**All boys**	**All girls**		**<13 y**	**≥13 y**		**<13 y**	**≥13 y**	
	**[n = 98]**	**[n = 141]**	**P- value**	**[n = 62]**	**[n = 34]**	**P - value**	**[n = 74]**	**[n = 67]**	**P-value**
**Misconception 1** ^**1]**^ **:**									
ISO-BMI*	4.1 %	16.3 %	0.003	3.2 %	5.9 %	0.533	1.4 %	32.8 %	0.000
WC**	4.1 %	11.5 %	0.043	3.2 %	5.9 %	0.533	0.0 %	24.2 %	0.000
**Misconception 2** ^**2]**^ **:**									
ISO-BMI*	6,1 %	6,4 %	0.935	4.8 %	8.8 %	0.440	9.5 %	3.0 %	0.116
WC**	16.3 %	20.9 %	0.381	17.7 %	14.7 %	0.703	26.0 %	15.2 %	0.115

^1]^Misconception 1: Perception of overweight without being measured as overweight/obese according to ISO-BMI and WC respectively. ^2]^ Misconception 2: Perception of non-overweight despite being measured as overweight/obese according to ISO-BMI and WC respectively

*ISO-BMI = age and gender-specific body mass index [BMI] for children up to 18 years. **WC = Waist Circumference

## Discussion

This study showed social inequality in overweight and obesity, especially among boys < 13 years, but not in regard to perception of overweight, which was commonly found among girls ≥ 13 years. These age- and gender-specific findings might contribute to a better understanding of the developmental pathways leading to weight-related disorders.

### Age and gender-specific social inequality in overweight/obesity

The higher prevalence of overweight and obesity found among younger children of low-SES parents, especially among those with low-SES mothers, could be explained by lifestyle characteristics in addition to strong hereditary predisposition [9]. In families and especially among mothers with lower SES, less emphasis on, as well as limited access to, healthy eating habits and physical activity may be important risk factors for developing overweight and obesity [5, 9, 22, 23]. In addition, since lower SES has been found to be associated with higher prevalence of overweight among adults [24], parental overweight/obesity may affect perception of their children’s weight and motivation for their children to avoid further weight gain [25, 26]. It has been suggested that overweight mothers in particular have greater difficulty assessing weight in younger children when using themselves as a reference point [27, 28]. The greatest maternal misperceptions of weight status among children were observed among those children categorized as overweight/obese [28]. One study shows that parents believe that being “chubby” at a young age is “cute” and that overweight in childhood is not a problem prior to adolescence [29]. Moreover, research suggests that some physicians may lack the confidence to identify childhood overweight and do not always inform mothers that their children’s weight is of concern [30].

In addition, younger children spend more time with their parents, especially their mothers. Parents shape social norms and serve as models for behavior, likely reflecting the values of their social class [31]. However, the type and extent of parental influence on the child will vary in accordance with the perceived maturity of the child [32]. Parental influence decreases, while peer influence increases as children grow older, for which reason parents are likely to have a stronger influence on health behaviors in younger children [32]. It is well known that girls mature earlier than boys, suggesting that parents have less influence on girls than on boys of similar age. The latter might explain why we only found social inequality to be an influential factor in younger boys, but not in younger girls.

Among boys ≥ 13 years, we only found a significantly higher BMI and WC when their fathers had a low level of education. Lower SES is associated with an increase in prevalence of overweight and obesity among adults in developed countries [24], suggesting that fathers with a low level of education in this study are at increased risk of being overweight themselves due to the social norms and behaviors of their social class. In addition to hereditary factors, adolescent boys may be at increased risk for overweight and obesity by adopting their fathers’ norms and lifestyle when they serve as role models [13]. This in turn could presumably lead to increased social inequality as reflected by overweight that continues into adulthood.

### No social inequality, but age and gender differences found in perception of overweight

From the perspective of obesity prevention, one positive finding is that perception of overweight among boys and younger girls was associated with actual overweight according to both ISO-BMI and WC, and not with paternal or maternal SES. This finding stands in contrast to those of Park, who reported underestimation of overweight among boys, especially from low-income households [14]. Misconception 2, in which overweight is underestimated, was not found in our study. In the WHO HBSC study [33], no significant relationship between perception of body weight status and family SES was seen in most participating countries, with the exception of a few countries and regions, especially in Western Europe and North America, where the perception of overweight/obesity was found to be related to low family SES [33]. Our results show a realistic perception of overweight even among children and adolescents from low-SES backgrounds that had a higher prevalence of overweight, thereby suggesting that the strongest predictor for body dissatisfaction is overweight prevalence, at least among boys and younger girls [34].

Meanwhile, boys going through puberty have a natural potential to become more muscular and develop broader shoulders after their growth spurt, which both contribute to the positive ideal of the male body. Boys ≥ 13 years may therefore develop a more positive self-image with age and pubertal development [35, 36], which could explain the lack of misconception 1 among boys.

At the same time, the increase in body fat that naturally accompanies puberty in girls conflicts with stereotypes of the ideal female body. Girls in puberty have therefore been shown to develop a negative perception of their body [37], confirmed strongly in this study by the high prevalence of misconception 1. The WHO HBSC study [33] also found that in many countries 15-year old girls were significantly more likely to report that they were too fat than 11-year olds. This age difference in perception of overweight was not found among boys neither in this study nor in the HBSC study [33]. Compared with their mothers, adolescent girls have a less realistic and more negative perception of their body weight status [38]. However, the female desire to be thin shared by both mothers and girls [39, 40] may have a negative impact on perception of the body among girls.

Meanwhile, a comparison of prevalence of overweight based on self-reports in the HBSC study and on measured ISO-BMI in the present study may indicate a reporting bias among Swedish girls [41]. The prevalence of overweight according to ISO-BMI based on self-reports in the HBSC study was 9 % among Swedish girls, compared with 15 % based on measured ISO-BMI in the present study. This difference is not seen between self-reported or measured ISO-BMI among boys [both 14 %] [33]. The incongruence between perception of the body and actual body size in girls, particularly at older ages, was corroborated in the present study by the findings of a lower OR for perception of overweight with regard to measured overweight according to ISO-BMI and WC. Also, the increased OR for perception of overweight for age ≥13 when adjusted for measured ISO-BMI, along with the high prevalence of misconception 1 among girls ≥ age 13, confirm that factors other than actual body size determine perception of overweight in adolescent girls.

### Strengths and limitations

In the present study, perception of body weight status was self-reported, but is nonetheless considered to be a strength since self-reported perception of overweight is a determinant for actual and future weight and health behaviors [42]. In our study, the self-reported perception of overweight was compared not only with actual body size according to anthropometric measurements, but also with the Swedish and international results of the WHO HBSC study performed the same year [33]. The latter compensated for the small study population regarding perception of body weight status, but also confirmed the importance of comparing physical measurements with self-reported weight and height, especially in girls. In boys, the comparison of self-reported weight and height in the HBSC study [33] and the measurements in this study led to the same prevalence of overweight and obesity, implying that there was no clear bias in the selection of participants regarding being overweight, despite the small study population.

Our small study population limited generalization of the results, and also allowed only classification into two SES groups. Another limitation was the fact that parental SES was self-reported and not obtained through high-quality register data for ethical reasons. In this study, levels of parental education and occupational status were treated as two separate indicators of SES, despite the fact that they correlate with each other to some extent. Consequently the results should be interpreted with caution. The same applies to the separate analysis for paternal and maternal SES, even though paternal and maternal impact on children and adolescents was a partial goal of this study.

The extent to which social inequality increases the risk for overweight/obesity, and affects age and gender specific differences in perception of overweight, requires further investigation through larger quantitative and complementary qualitative studies. Age and gender differences in awareness of social norms and behaviors for development of overweight and perception of overweight should receive special attention.

## Conclusions

The association between social inequality and overweight in adolescence in this study is age- and gender-specific. Low parental SES, especially among mothers, is associated with overweight among boys in childhood, while low paternal SES is associated with anthropometric measures in adolescence among both boys and girls. Gender differences, especially in perception of overweight, tend to increase with age, indicating that adolescence is a crucial period. A better understanding of the developmental pathways involved, including from a gender perspective, may help to identify ways to reduce the impact of social inequality on weight gain in adolescence and subsequent adult health.

In planning interventions to prevent overweight and obesity among children and adolescents, the interplay between parental influences and pubertal development processes related to overweight and perception of overweight among boys and girls should be taken into account.

## Abbreviations

BMI: Body Mass Index
CI: Confidence Interval
FORSS: Medical Research Council of Southeast Sweden
HBSC: Health Behavior in School-aged Children
HUBRO: Oslo Health Study
ISO - BMI: age and gender-specific BMI for children up to 18 years
ISO – BMI 25: age and gender-specific BMI for overweight defined by Cole et al. [19]
ISO – BMI 30: age and gender-specific BMI for obesity defined by Cole et al. [19]
OR: Odds Ratio
SD: Standard deviation
SES: Socioeconomic status
WC: Waist circumference
WHO: World Health Organization
